# Persistence of the Exotic Mirid *Nesidiocoris tenuis* (Hemiptera: Miridae) in South Texas

**DOI:** 10.3390/insects12080715

**Published:** 2021-08-10

**Authors:** Gabriela Esparza-Diaz, Thiago Marconi, Carlos A. Avila, Raul T. Villanueva

**Affiliations:** 1 Texas A&M AgriLife Research, 2415 East Highway 83, Weslaco, TX 78596, USA; thimarconi@gmail.com (T.M.); carlos.avila@ag.tamu.edu (C.A.A.); 2 Department of Horticultural Sciences, Texas A&M University, College Station, TX 77843, USA; 3 Department of Entomology, University of Kentucky Research & Education Center, 348 University Drive, Princeton, KY 42445, USA

**Keywords:** mirid, invasive pest, biocontrol agent

## Abstract

**Simple Summary:**

The Rio Grande Valley is one of the most productive agricultural areas in the U.S, located in the southernmost part of Texas. In October 2013, we detected an exotic species of plant bug occurring in this region. It was identified as *Nesidiocoris tenuis*, which had a phytophagous behavior on tomato plants in the absence of its preferred prey. We confirmed the species with morphological and genetic tests. We monitored populations of *N. tenuis* in its introduction phase in commercial fields and corroborated its establishment in research fields for three consecutive years. The presence of *N. tenuis* could establish a new relationship of trophic insects to produce vegetables in the Rio Grande Valley. However, it is unknown whether the presence of *N. tenuis* in the Rio Grande Valley can help control pests of economic importance, such as whiteflies in cotton, or become a pest on sesame, which is an emerging crop in this region.

**Abstract:**

The Rio Grande Valley is one of the most productive agricultural areas in the U.S and is located in the southernmost part of Texas. In October 2013, we detected an exotic plant bug, *Nesidiocoris tenuis* Reuter (Hemiptera: Miridae: Bryocorinae) occurring in the region. *Nesidiocoris tenuis* has zoophytophagous habits; however, in the absence of insect prey, it feeds on its plant hosts. After its morphological and genetic identification, this study monitored the population of *N. tenuis* in its introduction phase in commercial fields and corroborated its establishment in research fields for three years. Populations of *N. tenuis* were high during the fall and low during winter. This study found that *N. tenuis* populations were higher in tomato fields as compared to adjacent pepper, okra, and squash fields, indicating its host preferences during the introduction phase. Recurrent population growth patterns suggest that *N. tenuis* was established in Rio Grande Valley with permanent populations in tomato fields. In addition, *N. tenuis* populations were affected by tomato cultivar selection and by plastic mulch color. The presence of *N. tenuis* could establish a new trophic insect relationship for vegetable production. However, it is unknown if the presence of *N. tenuis* may help to control pests of economic importance, such as whiteflies in cotton, or become a pest on sesame, an emerging crop.

## 1. Introduction

Plant bugs in the family Miridae have different feeding habits including phytophagy, zoophagy, and zoophytophagy. The diversity of feeding habits allows them to feed on several hosts and prey, especially those species with zoophytophagous behavior, giving them the ability to thrive and colonize a broad range of environments. One of the most abundant Miridae in the European Mediterranean area is *Nesidiocoris tenuis* (Hemiptera: Miridae: Bryocorinae), previously *Cyrtopeltis* or *Engytatus* [1,2,3,4,5]. Several species are known as tomato bugs, including *Engytatus modestus* [6], *Engytatus varians* [7], and *N. tenuis* [8]. Additionally, *N. tenuis* has been called green tobacco capsid [9], tobacco leaf bug [10], tomato mirid, and tomato suck bug [11].

*Nesidiocoris tenuis* has spread widely into new geographic areas since its original description in the Canary Islands, the Philippines, and several countries in the Middle East, where this species is considered exotic [12,13,14,15]. Currently, *N. tenuis* has been recorded worldwide except in some countries in South America, Northern Europe, and Russia [16]. The colonization of the new areas by *N. tenuis* may be due to changes in environmental conditions favorable to its establishment to intentional introductions (*N. tenuis* is commercially available in Europe), or unintentional transporting in international commerce. It is hypothesized that a species of beetle could cross the desert along a new railway route, as indicated by temporal and spatial analysis of the findings of Harmonia axyridis in Kazakhstan and Kyrgyzstan after the construction of the Turkestan–Siberia railway [17]. Furthermore, a species of fly (*Hermetia illucens*) may have spread along the Black Sea coast by human maritime flow [18]. The success of an invasion of *N. tenuis* to new areas could depend on its invasiveness and the vulnerability of the agroecosystem receptor [19]. The latter has resulted in the geographical expansion of *N. tenuis* that may lead to a potential invasive behavior but with possible resulting benefits. In the case of an intentional introduction, i.e., France [12], *N. tenuis* represented an ecological risk; however, this insect provided potential benefits as a predator in the greenhouse production of tomatoes (*Solanum lycopersicum* L.) [20].

When abundant prey is present, *N. tenuis* acts as a predator feeding on small arthropods [20,21]. However, in the absence of insect prey, its phytophagous feeding behavior increases, causing high flower abortion rates and necrotic rings on stems and leaf petioles that can negatively impact the plant growth in tomatoes [4,20,22]. *Nesidiocoris tenuis* has been utilized in IPM programs as an effective natural enemy to control several pests in tomato production. In Spain, it has been successfully used to control whiteflies (*Bemisia* spp., Hemiptera: Aleyrodidae) in greenhouse-grown plants [20]. Similarly, *N. tenuis* is useful in the biocontrol of the tomato borer *Tuta absoluta* Meyrick (Lepidoptera: Gelechiidae) [23,24,25]. *Tuta absoluta* is native to South America and is considered a key invasive species of tomato in Europe. In contrast, in two studies, *N. tenuis* has also been reported as a potential crop pest [16,23,26]. Therefore, the status of *N. tenuis* as a beneficial insect or pest is controversial where this species is native. 

*Nesidiocoris tenuis* has been reported in the U.S from Florida, California and New Mexico [27], and CABI International has reported sporadic detections of *N. tenuis* since 1971 [28]. However, until our findings in 2013, and to this date, it has not been reported in Texas as an established invasive or predatory species yet. Currently, *N. tenuis* is one of the polemical biocontrol agents because of the balance between its benefits as a predator and the plant-feeding damage it causes [4,29]. The Rio Grande Valley in South Texas is an agricultural area with economic significance that transborder socio-cultural and territorial circumstances; it is in a floodplain draining into the Rio Grande River with a subtropical climate. In October 2013, we detected an abundant presence of *N. tenuis*. This document reports the morphological and molecular identification, hypothesizes its introduction, describes its expansion and persistence phases as a non-native species, and discusses the potential significance of this species on vegetable crops grown in the region.

## 2. Materials and Methods

### 2.1. Identification of Exotic Mirid as N. tenuis in South Texas

#### 2.1.1. Morphological

The initial infestation of adults and nymphs was conducted in October 2013 in an experimental tomato field in Edinburg, TX, USA. We collected specimens from tomato plants that were in the flowering and fruit formation stages and sent them to Dr. Thomas J. Henry (USDA-ARS Systematic Entomology Laboratory, Beltsville, MD, USA) for taxonomic identification.

#### 2.1.2. Molecular

*Nesidiocoris tenuis* DNA barcoding was conducted. Specimens collected from Rio Grande Valley fields and stored at −80 °C were used as a putative material, while commercial samples from Spain were a control positive and the tomato-potato psyllid (*Bactericera cockerelli*, Sulc Hemiptera: Sternorhyncha) was an outgroup control. Ethanol (70% vol/vol)-preserved *N. tenuis* specimens were collected by Villanueva (co-author of this report) in June 2014 in Valencia, Spain, and were utilized for molecular analysis. The laboratory of Plant–Insect Interactions in the Department of Horticultural Science, Texas A&M University–AgriLife at Weslaco conducted the purification of amplified PCR products. Legs from a total of 4 insects were used to isolate DNA using the CTAB method [30]. Barcoding of universal degenerative primers, (*LepR1* 5′-TAA ACT TCT GGA TGT CCA AAA AAT CA-3′ + *MHemF* 5′-GCA TTY CCA CGA ATA AAT AAY ATA AG-3′) [31], and *N. tenuis-*specific primers, (*Nt*_*COI_F* 5′-ACT TCA GGG TGC CCA AAG AA-3′ + *Nt*_*COI_R*-5′-TGT GAA AAG GGG TAT CCA CCA-3′), was used to amplify the mitochondrial *Cytochrome c Oxidase subunit I* (*COI*). A *Nesidiocoris tenuis* particular primer set was developed using the *COI* consensus sequence from complete mitochondrion genome (GB JQ806057.1) at the National Center for Biotechnology Information (NCBI) using Primer-BLAST [32]. The PCR incubation with degenerative primers was performed under the following conditions: 2 min at 95 °C; five cycles of 40 s at 94 °C, 40 s at 45 °C, and 1 min at 72 °C; 35 cycles of 40 s at 94 °C, 40 s at 51 °C, and 1 min at 72 °C; 5 min at 72 °C; held at 4 °C. The *Nt_COI* reactions were performed at 95 °C for 3 min, 34 cycles of 95 °C for 30 s, 55 °C for 30 s, and 72 °C for 1 min, and finally, an extension at 72 °C for 5 min held at 4 °C. PCR products were separated on 1% agarose stained with GelRed (Biotium, Fremont, CA, USA). Amplicons were excised from the gel and cloned using pGEM T-easy vector (Promega, Madison, WI, USA) following the manufacturers’ methods and sent to Alpha Biolaboratory, Inc. (Saratoga, CA, USA) for sequencing. Data with a Phred score lower than 20 was trimmed using Codon Code Aligner v 6.02 (CodonCode Corporation, Centerville, MA, USA) and the resulting sequences were BLAST against the (NCBI) database for DNA barcoding.

### 2.2. Introduction Phase of N. tenuis

After confirming the species, we detected a localized invasion on several vegetables. The natural incidence of *N. tenuis* was concentrated on an experimental tomato field in October 2013 and adjacent fields including bell pepper (*Capsicum annuum* L.), okra (*Abelmoschus esculentus* L.), and squash (*Cucurita pepo*). We record the populations in tomatoes the other crops. Tomato plants were established on 0.04 hectares with 18 rows and 18.8 m in length. The field was divided into six rows per replication with two rows of corn as an intercrop. Each replication of six rows had three plots with 6.1 m length, resulting in nine plots. A plot per replication was randomized for the estimation of *N. tenuis*. Tapping was used to tally nymphs, and adults were tallied by shaking the plant foliage over a white plastic surface of a shoebox lid (21 cm wide by 35 cm long, or 735 cm^2^). Samples were conducted in the morning to minimize mirids from moving away before counting them. This active population in tomatoes was monitored on 18, 21, 25 October 2013, and 6 and 12 November 2013. In the initial detection on 18 October 2013, *N. tenuis* were easily observed on tomatoes. Tallies of *N. tenuis* were extended to adjacent fields of bell pepper, okra, and squash. *Nesidiocoris tenuis* populations were tallied by visual inspection in situ in an area of ~2000 m^2^/vegetable. All vegetables were in areas with irrigation tape and two rows of corn plants as an intercrop as a wind barrier. Inspections consisted of aleatory five plants/replication, and four replications/vegetables to estimate the number of adults and nymphs per plant. The plants were in the first stages of flowering and fruit initiation, except okra plants, which were in first fruit development. Descriptive statistics analyzed the insect incidence across crops by inspection date.

### 2.3. Expansion Phase of N. tenuis

*Nesidiocoris tenuis* populations were monitored in three commercial tomato fields across the Rio Grande Valley in 2014. Monitored plots included two commercial tomato fields located in Pharr (26.066558°; −98.202126°) and Edinburg (26.265800°; −98.092583°) Hidalgo Co., TX, USA and in a greenhouse in San Benito, Cameron Co., TX, USA (26.066324°; −97.575754°). In the first-mentioned field, three replications of 150 plants in groups of 10 plants were selected per replication. Tallies of *N. tenuis* were in random replications and carried out using the tapping method described above.

### 2.4. Persistence Phase of N. tenuis

Natural population fluctuations of *N. tenuis* were monitored in two tomato growth seasons from December 2013 through January 2015. The experimental field was at the Texas A&M AgriLife Research and Extension Center in Weslaco (26.159244°; −97.960701°). The experimental plot consisted of 300 tomato breeding lines for the fresh tomato market. Tomato plants were transplanted in a row bed with a 0.61 m spacing method in a 2 m width. Two sorghum rows divided plots running parallel to the 300 tomato plants per cultivar as an intercrop wind barrier. In this field, 150 plants were used exclusively to tally *N. tenuis,* and the second group of 20 random plants was destined to *B. tabaci*. The *N. tenuis* population was estimated by the tapping method described above. Simultaneously, the *B. tabaci* populations were recorded by counting the number of nymphs per compound leaf. The monitoring was performed during the fall-winter seasons of 2013–2014 and 2014–2015 every two weeks from the vegetative to the harvest initiation period.

### 2.5. Evaluation of N. tenuis Populations in Tomato Cultivars

To evaluate *N. tenuis* populations on different tomato genotypes, a study was established in the experimental field at the Texas A&M AgriLife Research and Extension Center in Weslaco using 900 plants. Tomato plants were transplanted in a row bed with 0.3 m spacing and a zig-zag method in a 2 m wide row. Three cultivars were utilized including cv. T5 (provided by Kevin Crosby in the Texas A&M Breeding program). In addition, two other varieties with an Organic Materials Review Institute certificate (Charger F1 from Johnny’s Selected Seeds, Winslow, ME; and Crimson Sprinter from High Mowing Organic Seeds, Wolcott, VT, USA) were used. Two sorghum rows were used as crop break plots running parallel to the 300 tomato plants per cultivar as an intercrop wind barrier. Tallies of *N. tenuis* were done as described above (tapping foliage). The population of *B. tabaci* was monitored as nymphs per leaf using a stereoscope. Both populations were estimated on six dates from December 2013 through February. A line of the plants per row was exclusive for *N. tenuis* counting, and another line of the plants was designated to *B. tabaci* incidence. Evaluations started in the vegetative stages (20–25 days after emergence) and were finalized when the plant was at harvest initiation.

### 2.6. Evaluation of N. tenuis in Tomato Grown Using Different Plastic Mulch of Different Colors

In a second group of the tomato plants, we studied *N. tenuis* incidence on three plastic mulch colors that included white, silver (known as reflective), and black colors using the cv. Crimson Sprinter. The commercial plastic cover was 0.0254 mm thick and made of high-density polyethylene plastic with a width of 1.8 m. Tomato plants were transplanted in a double-row bed with the zig-zag spacing method in a row with a 2 m width. A line of the plants was exclusive for *N. tenuis* counting, and the other line of the plants was designated to *B. tabaci* records. Each mulch color had a total of 300 plants using two rows of sorghum as described above. *Nesidiocoris tenuis* counts were performed as described above during the same dates. 

### 2.7. Plant Damage per Tomato Variety

Under controlled conditions, we studied if there was a tomato varietal response to plant damage by *N. tenuis*. *Nesidiocoris tenuis* feeding per cultivar was estimated in two varieties in the absence of *B. tabaci*. Tomato plants of the cv. Lance and UC82 were the varieties grown under controlled conditions in a growth chamber at 25 °C ± 2; 70% HR ± 10 and 16:8 h L:D photo phase. Three hundred specimens per replication were placed in screened cages (to prevent *N. tenuis* escape) containing pest- and disease-free plants using an insect cage. Non-infested plants were kept under the same conditions as the control, and two replications per treatment were performed. After one month, the plant growth was evaluated as a dichotomous variable with two possible values measured as health or injuries caused by *N. tenuis*.

### 2.8. Geo-Reference, Environmental Data, and Statistical Analysis

Field locations were registered by a global positioning system using a GARMIN^®^ GTM35 unit (Garmin International, Olathe, KS, USA). The Climate Dataset was produced and archived at NOAA’s National Climatic Data Center (NCDC) of the station GHCND: USC00419588, Weslaco, TX US. Climate data for LGV was acquired from Climate Data Online of NCDC’s archive historical weather along with climate data in addition to station history information. These data included daily measurements of maximum (*T*_max_) and minimum (*T*_min_) temperatures from 9 October 2013 to 16 March 2015. The daily temperature trends for maximum (*T*_max_ Trend) and minimum values (*T*_min_ Trend) were calculated by fitting with distance-weighted least squares (DWLS) [33]. The effects of the mulch color and tomato cultivar on *N. tenuis* incidence that occurred naturally were analyzed independently. The mulch color and tomato cultivar were independent variables, while *N. tenuis* and *B. tabaci* populations were dependent variables in a randomized complete block design for each factor. Data analysis of insect populations was transformed using the square root (x + 0.5) before the analysis for normality [34]. Statistical significance of differences among treatments was determined using either analysis of variance (ANOVA) or a Fisher’s LSD for mean separation. Statistical analyses were performed with Statistica 12 software (StatSoft Inc., Tulsa, OK, USA).

## 3. Results

### 3.1. Identification of Exotic Mirid as N. tenuis in South Texas

#### 3.1.1. Morphological

The collected putative insect specimens during our first sight location in 2013 were morphologically identified by Dr. Thomas J. Henry (Systematic Entomology Laboratory, USDA-ARS) as *Nesidiocoris tenuis*. Vouchers of collected specimens were deposited in the research entomology collection of the National Museum of Natural History in Washington D.C. and the Texas A&M University insect collection at College Station, TX, USA.

#### 3.1.2. Molecular

**Figure 1:**
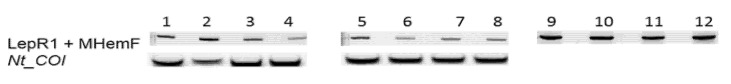
Amplification of the mtCOI gene using *N. tenuis* degenerate and specific DNA barcoding primers. *Nesidiocoris tenuis* from Spain (samples 1–4) and Texas (samples 5–8), and outgroup *B. cockerelli* (samples 9–12).

**Figure 2:**
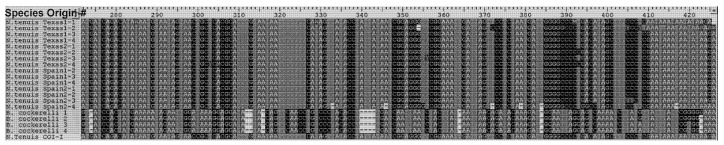
Alignment of sequenced mtCOI amplicons.

The DNA barcoding performed confirmed the morphological identification of *N. tenuis* via highly conserved mitochondrial cytochrome c oxidase subunit I (COI) gene. The initial test found the best degenerative primer combination for amplification of the COI gene among the list described by Park et al. in 2011 [31]. The primer combination LepR1 + MHemF was found to have the most optimized amplification among all the samples (data not shown). Generated sequences (Figure 1) were edited to remove the lower quality data using Codon Code Aligner v6.02 (CodonCode Corporation, Centerville, MA, USA) and compared against the National Center for Biotechnology Information (NCBI) database. BLAST results indicated that the identity of all specimens collected in Texas and positive controls from Spain were *N. tenuis* (>99.15% and >98.87% match identity, respectively; Supplemental File S1) and the outgroup, as expected, was B. cockerelli (>97.11% match identity; Supplemental File S1). For future specimen identifications, the consensus sequences of *N. tenuis* were used to design specific *N. tenuis* COI primers (Nt_COI). PCR amplicons using Nt COI primers were only obtained when DNA was isolated from *N. tenuis* as a template, confirming the probe specificity for *N. tenuis* mitochondrial COI gene (Figure 2).

### 3.2. Introduction Phase of N. tenuis

**Figure 3:**
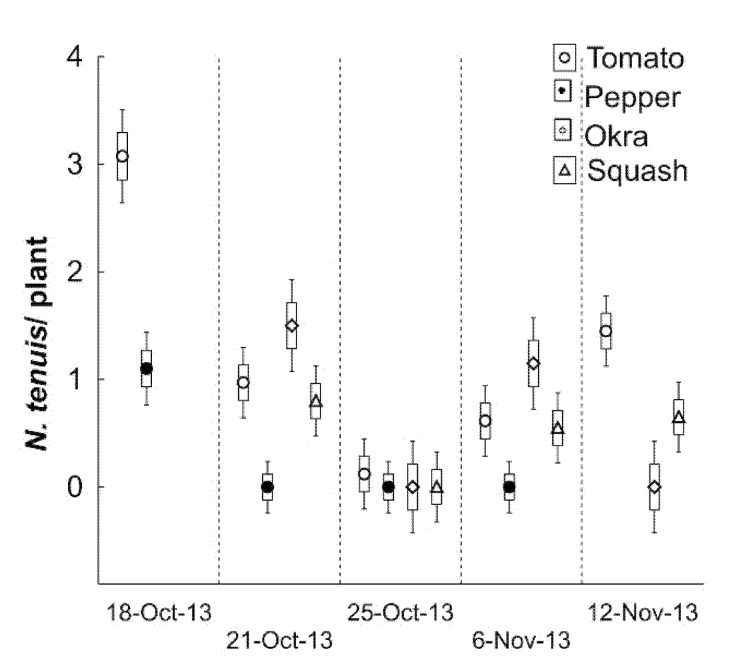
Introduction phase of *Nesidiocoris tenuis* on vegetable fields in Edinburg, TX, USA. Plot data points shown means, standard errors, and 95% confidence intervals of *N. tenuis* on tomato, pepper, okra, and squash fields.

**Figure 4:**
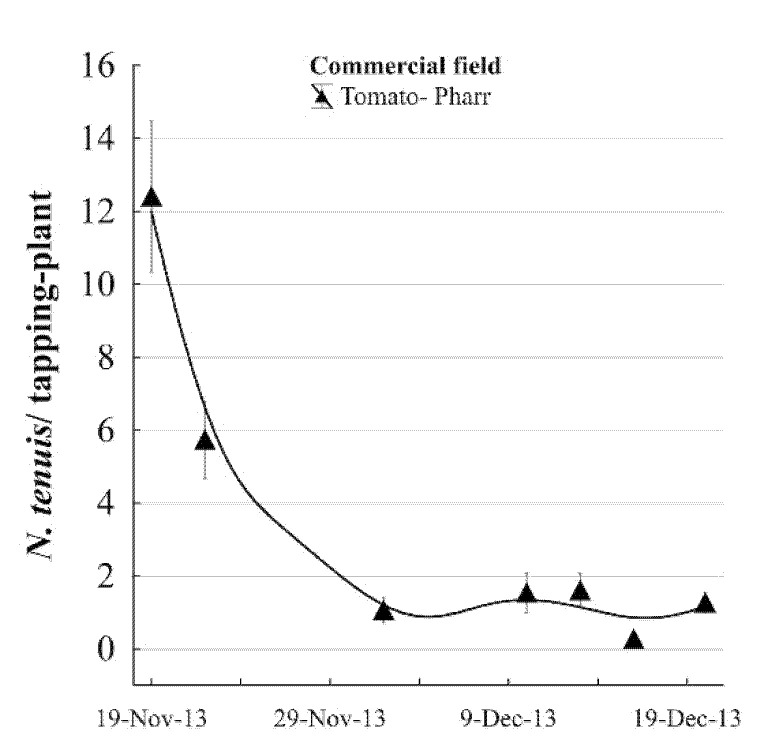
Mean (±SE) population of *Nesidiocoris tenuis* on tomato plants in a commercial field located at Pharr, Hidalgo Co. TX, USA. The moving mean curves shown with lines were fitted by a distance-weighted least squares method.

Initially, *N. tenuis* populations were found in tomato plants in the experimental field described above, when the daily *T*_max_ Trend was 28.5 °C. In addition, *N. tenuis* infestations on tomatoes were detected in five sampling dates until mid-November. The highest population occurred at the beginning of the detection, and by the end of October, *N. tenuis* declined when *T*_min_ Trend was under 15.8 °C. By the first days of November, the *N. tenuis* population had a few recuperations (Figure 3). In addition, *N. tenuis* was observed on pepper, okra, and squash, although its incidence was lower on these crops than on tomato plants (Figure 4). In this introduction phase, *N. tenuis* had a polyphagous behavior as prey was not recorded. Tomato in this field showed typical feeding damage by an active population of *N. tenuis* in the plants. Symptoms included stunted growth and necrotic lesions in the meristems. 

### 3.3. Expansion Phase of N. tenuis

**Figure 5:**
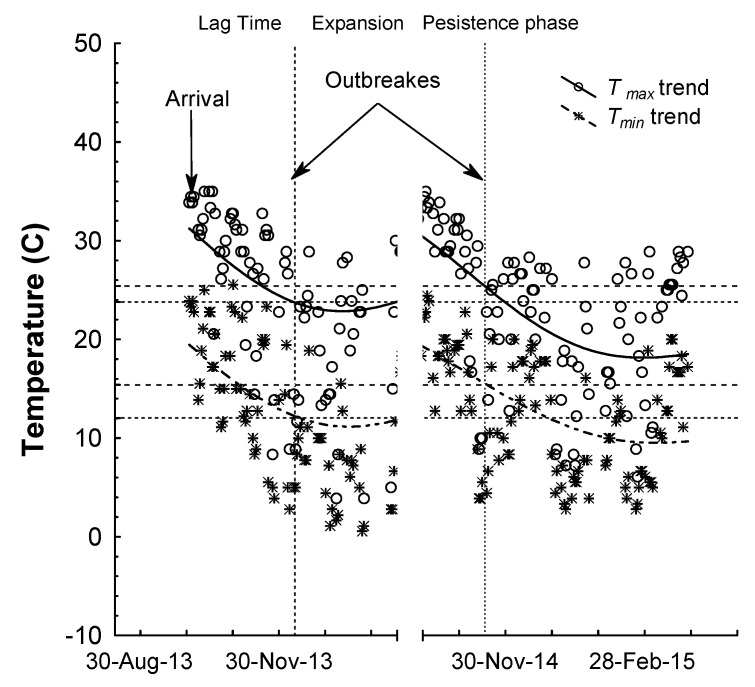
Daily data of maximum (*T*_max_) and minimum (*T*_min_) temperatures in °C from 9 October 2013 to 16 March 2015. During the lag time/expansion/persistence phases of *N. tenuis*. Daily *T*_max_ (circle) and *T*_min_ (asterisk) are shown. The moving mean curves were fitted by a distance-weighted least squares method of daily temperature trends for *T*_max_ and *T*_min_. Lines with the intersection on the Y-axis show the values of temperature trend lines of two *N. tenuis* outbreaks in 2013 and 2014. Data are from the Climate Dataset, NOAA’s National Climatic Data Center (NCDC) of the station GHCND: USC00419588, Weslaco, TX, USA.

Our first detection of the *N. tenuis* in a commercial tomato field occurred during the fall of 2013 in Pharr, TX, USA. This was 15 km from the first point of *N. tenuis* detection on the experimental field. *Nesidiocoris tenuis* populations were high during the first weeks of November while tomatoes were flowering and the weather was still warm. Then, the populations decreased as temperatures decreased (Figure 4 and Figure 5). In the organic commercial farm in 2013, there was total yield loss due to a great abundance of *N. tenuis* reaching over 25 ± 1.4 specimens per plant. As tomato is not planted in the Rio Grande Valley from May to September, *N. tenuis* was found in a greenhouse in San Benito, TX, USA. However, in this case, *N. tenuis* was not associated with a yield reduction; instead, it may have contributed to the control of whiteflies. In the commercial fields, the incidence of *N. tenuis* covered 363 km^2^ in the fall seasons of 2013–2014. We detected a rapid geospatial dispersion of *N. tenuis* on commercial tomato fields in South Texas.

### 3.4. Persistence Phase of N. tenuis

**Figure 6:**
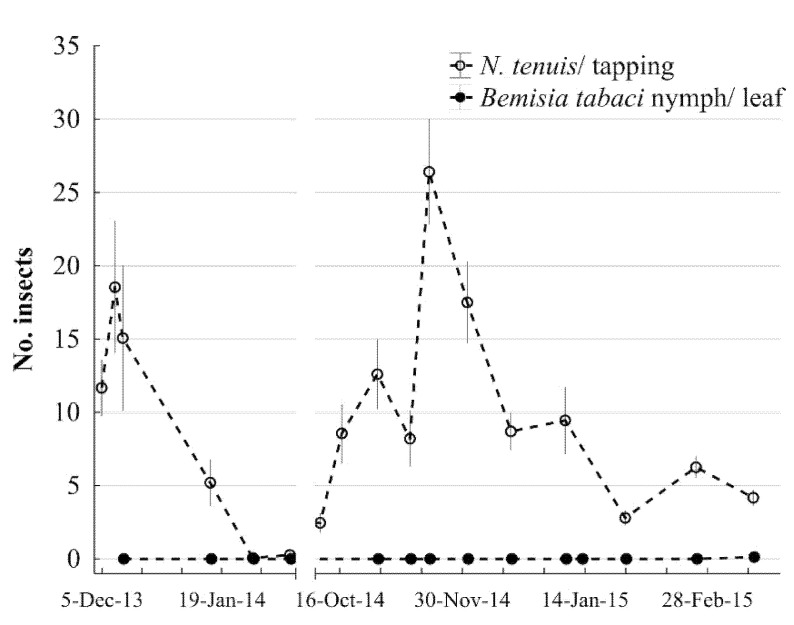
Seasonal incidence of *N. tenuis* and *Bemisia* sp. on tomato breeding fields. Mean (±SEM) of *N. tenuis* per plant (tapping) and *Bemisia* sp. per tomato leaf on the experimental field at Weslaco, TX, USA and *Bemisia* sp. per tomato leaf on the experimental field at Weslaco, TX, USA.

The *T*_max_ ranged from 25.4 °C to 23.8 °C and the *T*_min_ ranged from 15.4 °C to 12 °C (Figure 5). In the mid-winter 2013, *N. tenuis* numbers reached the lowest numbers, probably caused by the drop of daily temperatures under 10 °C (25 January 2014). During the same period, *B. tabaci* was not found while conducting the *N. tenuis* monitoring, and it was not detected until 17 March 2015 (0.08 insect/leaf) (Figure 6). However, *N. tenuis* was also affected by temperatures above 30 °C (Figure 5) when *N. tenuis* populations were low. The seasonal monitoring in the experimental field located at the Weslaco-Texas A&M AgriLife Research (described above) and Extension center showed that *N. tenuis* populations’ natural fluctuation had two outbreaks in December 2013 and November 2014 (Figure 6).

### 3.5. Evaluation of N. tenuis Populations in Tomato Cultivars

**Figure 7:**
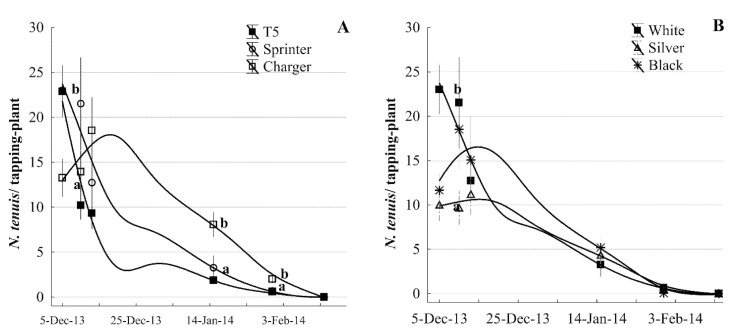
Effect of tomato cultivar and plastic color cover on *N. tenuis* population (mean ± SE): (**A**) Cultivar evaluation and (**B**) row mulch color evaluation. Means followed by different letters on a date are significantly different (*p* ≤ 0.05, ANOVA and Fisher’s HSD). The moving mean curves shown with lines were fitted by a distance-weighted least squares method.

The effect of tomato cultivars was significant in the incidence of *N. tenuis*. Out of the three varieties tested, the cv. Charger F1 showed an increase in the *N. tenuis* population after the first evaluation date and mean numbers were significantly greater on 15 January 2014 (F2, 72 = 7.8267; *p* = 0.000840) and 31 January 2014 (F2, 72 = 10.93; *p* = 0.000072) compared with the T5 and Sprinter cultivars, where *N. tenuis* populations decreased after the first evaluation (Figure 7A). 

### 3.6. Evaluation of N. tenuis in Tomato Grown Using Different Plastic Mulch of Different Colors

In our cultivar trial, the effect of plastic mulch color in planted rows was significant on the *N. tenuis* population only during the initial period on 5 December (Figure 7B). *Nesidiocoris tenuis* counts were higher on white mulch followed by black and silver with the lowest (F2, 42 = 10.4109; *p* = 0.000213). Despite our exhaustive examination and tallying 900 leaves, *B. tabaci* nymphs were absent during the entire monitoring period (Figure 7B); in contrast, *N. tenuis* total tallies reached a total of 2428 specimens.

### 3.7. Plant Damage per Tomato Variety

**Figure 8:**
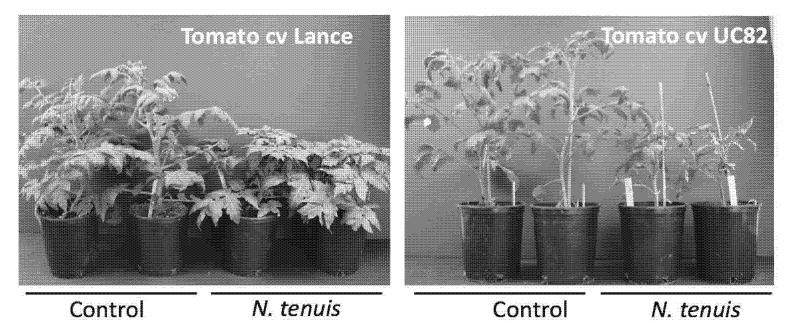
*Nesidiocoris tenuis* visual damage in the cv. Lance and cv. UCB2 tomatoes. Plant growth was visually inspected one month after infestation by comparing with non-inoculated plants. In both varieties, *N. tenuis* stunted plants and decreased foliage.

We corroborated that the mirid was causing plant damages, and the Lance and UC82 tomato cultivars infested with 300 insects had symptoms like the ones observed in the experimental area in Edinburg one month after release in cages. Plant growth was stunted as compared to non-infested plants with necrotic ring lesions on the stems (Figure 8). This finding suggested that *N. tenuis* was the causative agent of these injures and symptoms observed in the field.

## 4. Discussions

The *Nesidiocoris tenuis* evaluated in this study had morphological characteristics, such as black spots or bands, that stood out against the light green background color, and a black edge of the head [35]. The head, with clypeus, eyes, and a band on the posterior margin was black, which is typical of the species [35]. Using these patterns, a dichotonomous key, and molecular analyses between Texas specimens and those from Valencia, Spain, the identity of this mirid was confirmed. 

The presence of *N. tenuis* populations in several vegetable fields and constant incidence in tomatoes from 2013 to 2015 and the years after this date suggested that this exotic species is established in the Rio Grande Valley. In this study, we may have found the period of *N. tenuis* arrival (introduction phase), and its geographical expansion range in an area of 363 Km^2^, until its persistence or establishment phase with a natural fluctuation population and two registered outbreaks of consecutive fall seasons (2013 and 2014). The introduction, expansion, and establishment phases for *N. tenuis* are based on the incidence patterns, geographic expansion range, and natural fluctuations of populations through two growing tomato seasons (Figure 3, Figure 4, Figure 6 and Figure 7). We recorded that this insect species had a host plant preference for tomatoes vs. peppers, which were present in adjacent experimental fields, but with undetectable damage on okra and squash. In these two latter vegetables, *N. tenuis* was found concentrated in flowers, possibly consuming pollen. 

It is unknown when and how *N. tenuis* became established in the Rio Grande Valley. Lately, *N. tenuis* has expanded to the states of Sinaloa, Sonora, and Baja California Sur [36]. We can speculate on how *N. tenuis* arrived at the Rio Grande Valley with the following hypotheses: (a) it may be a result of environmental changes carried by winds from the Caribbean or Florida, (b) it may be an accidental introduction throughout the increased of international trade in the Rio Grande Valley, and (c) it could be natural migration of the insect. Trade upsurged in this region since 2010, when several international bridges and environmental storage facilities were developed to move fresh produce from Mexico to the U.S., surpassing Nogales, Arizona, the previous main port of entrance of trucks (L. Ribera, personal communication). In addition, other invasive pests were detected in the Rio Grande Valley by authors of this manuscript, including the South American palm weevil (*Rhynchophorus palmarum,* L Coleoptera: Cuculionidae) [37] and the Bagrada bug (*Bagrada hilaris* (Burmeister) Hemiptera: Pentatomidae [38]) in 2015. *Nesidiocoris tenuis* is now widely distributed in the world through human-assisted introductions, including in Africa, Australia, southern Asia and Europe, many of the Indian and Pacific Islands, and the Western Hemisphere [36]. Near to our area of study, the Rio Grande River separates Mexico and U.S, and across this river is located the state of Tamaulipas. In this state, there were almost 1500 hectares of tomatoes grown in commercial fields in 2014 [39] and 205 hectares of greenhouses [40]. In these greenhouses, an indeterminate growth of tomatoes was cultivated over the entire year in locations not further than 5 km from the U.S. border. In this type of crop system, *N. tenuis* is well adapted and used in Spain [41]. On the other hand, in the U.S, large numbers of tomato fields are planted in September every year. On either side, growers might have imported this predaceous species ignoring the regulations to use exotic natural enemies. Once in this region (in either side of the Rio Grande River), *N. tenuis* might have expanded and had an easy adaptation to a new ecological habitat. However, two authors of this report conducted scouting in other regions in Mexico, including the states of Veracruz (Manlio Fabio Altamirano and Banderilla) and Coahuila (Saltillo), and *N. tenuis* was not found on tomato fields and greenhouses (Villanueva and Esparza-Diaz, unpublished).

The presence of *N. tenuis* as exotic species might have established a new trophic insect interaction, and *N. tenuis* can be a doubtful advantage for growing tomatoes or other crops in the Rio Grande Valley. The recurrent population growth patterns suggest that *N. tenuis* was established in Rio Grande Valley with high invasibility of the agroecosystem receptor [19]. It may be beneficial for tomato production, as it may help to control populations of whiteflies (*Bemisia* sp.), one of the most important pests in tomato production in the Rio Grande Valley. In our case, we examined 900 tomato leaves exhaustively using a stereomicroscope, and *B. tabaci* nymphs were absent in all the cultivars; whereas the *N. tenuis* population reached a total of 2212 specimens across all the dates (Figure 7). This *N. tenuis* population and absence of *B. tabaci* suggested that *N. tenuis* might have been preying on *B. tabaci*. For this reason, *N. tenuis* is used as an effective biocontrol agent in greenhouse tomato production in Mediterranean countries (i.e., Spain, Egypt, and Turkey; [14,25,42]). Its use in those locations was reported as a success for control of *Bemisia* sp. and *T. absoluta* eggs. The USDA-APHIS has *T. absoluta* in the risk list of invasive species. In India, *N. tenuis* is one of the major pests of sesame (*Sesamum indicum*, Pedaliaceae), and insecticides are used for its control [43]. In the Rio Grande Valley, between 1000 and 5000 acres of sesame were planted in 2014–2015; in these sesame fields, farmers conducted at least one application of insecticides to control this insect [38]. Even in tomatoes, *N. tenuis* may cause crop damage that eventually results in yield loss. Sanchez [41] indicated that the population density of *N. tenuis* and the presence or absence of *Bemisia* sp. prey determines the crop damage level. For example, yield loss depends on *Bemisia* sp. abundance and can increase when the *N. tenuis*: *Bemisia* sp. ratio is >0.168. Therefore, yield reduction is caused when the *N. tenuis* population reaches its peak and the prey populations decline [29]. In the future, given the different conditions to grow tomato plants in the Rio Grande Valley (open fields vs. greenhouse in the Mediterranean countries), close monitoring of *N. tenuis* and its prey populations will be required to determine the potential role of this insect as a pest or as a potential biological control agent in the specific field.

Our results on tomato cultivars might have contributed to a preliminary preselection for the tolerance of tomato crops to *N. tenuis*; however, it is unknown if these results were the effect of antixenosis (insect host deterrence) or antibiosis (effects on fecundity and or survival). Regardless of the resistance mechanisms, our data showed that the Charger cultivar had large numbers of *N. tenuis* compared with the T5 and Sprinter cultivars. Therefore, cultivars and use of mulch can be used as a potential strategy to reduce *N. tenuis* populations. In addition, silver and white mulch color appeared to have an impact on *N. tenuis* infestation. The synergy of *N. tenuis* with the color of plastic mulch has not been studied or explained in other field conditions. However, insect pests could be affected by the color of the mulch, resulting in a smaller population, as is the case with aphids, where it was shown that black or white plastic mulch [44] and reflective mulch [45] affected them. However, more extensive evaluations are needed to validate this strategy. 

During the *N. tenuis* outbreaks in December 2013 and November 2014, the optimal temperatures for *T*_max_ and *T*_min_ were in the range from 23.8 °C to 25.4 °C, and from 12 °C to 15.4 °C, respectively (Figure 5). A study on *N. tenuis* adults found that this insect was susceptible to cold or high temperatures. It can lose the locomotor function at 4.0 °C and enter into a very cold coma at a significantly lower temperature (0.3 °C) [46], and it can also lose the ability to walk when the temperature is >43.5 °C and enter into a heat coma at 46.6 °C [46]. A study of eggs developed to adulthood showed that the highest mortality at 14 °C [47]. In a high temperature of 40 °C, *N. tenuis* is unable to develop and barely can reproduce [48]. In addition, Sanchez [41] reported that the optimal temperature for *N. tenuis* development oscillates between 20 °C and 30 °C; temperatures that are common in the Rio Grande Valley, where *N. tenuis* populations can thrive. *Nesiocoris tenuis* may invade and be established in most of the states around the Gulf of Mexico where the environment is favorable. 

Finally, the presence of *N. tenuis* led us to ask the following questions. (1) Will *N. tenuis* become permanently established in the Rio Grande Valley? (2) Will insecticide applications or other control strategies be necessary to control *N. tenuis*? (3) What will be the effect of *N. tenuis* on controlling whiteflies and other pests of tomatoes and several other vegetables grown in the region? For questions 1 and 2, the answer is that *N. tenuis* has been present since 2013. Since 2016, detections were recorded on sesame, requiring applications of insecticides to avoid pods or stunting plants (D. Sekula, personal communication, 22 April 2021). Although no reports are being associated with the presence of *N. tenuis* in other regions adjacent to the Rio Grande Valley, Hughes et al. [46] hypothesize that *N. tenuis* cannot survive cold temperatures and can have a limited permanent incursion to the northern areas in the U.S. However, according to our results, *N. tenuis* could have a positive result due to its ability to maintain an extremely low whitefly population in tomatoes. *Nesidiocoris tenuis* may continue to foray into other crops searching for prey such as whiteflies in cotton. Only in 2020 was *N. tenuis* observed in a whitefly-infested cotton field (D. Sekula, personal communication, 22 April 2021). Still, more information is required to address the impact of this invasive in the Rio Grande Valley and states around the Gulf of Mexico that can provide an environment suitable for the establishment of this insect. 

## 5. Conclusions

In October 2013, we detected an exotic plant bug, *Nesidiocoris tenuis,* in the Rio Grande Valley, the southernmost region of Texas. Here, we report for the first time its morphological and genetic identification on established populations in southern Texas. *Nesidiocoris tenuis* surpassed the introduction phase in commercial fields to its establishment in experimental fields, commercial fields, and greenhouses from 2013 to 2016. This study found that *N. tenuis* populations were higher in tomato fields as compared to adjacent pepper, okra, and squash fields, indicating its host preferences during the introduction phase. Persistent population growth patterns prove that *N. tenuis* was established in Rio Grande Valley with permanent populations in tomato fields. We found that *N. tenuis* populations can be affected by tomato cultivar selection and by plastic mulch color. *Nesidiocoris tenuis* may have established a new trophic insect relationship for vegetable production. *Nesidiocoris tenuis* may help to control pests of economic importance, such as whiteflies in cotton or tomatoes.

## Supplementary Materials

The following are available online at https://www.mdpi.com/article/10.3390/insects12080715/s1, File S1: Samples of mtCOI sequences and BLAST scores.
