# Supplementary material for: Persistence of the Exotic Mirid Nesidiocoris tenuis (Hemiptera: Miridae) in South Texas
Source: Insects. 2021 Aug 10;12(8):715. doi: 10.3390/insects12080715 (PMC8397022; doi:10.3390/insects12080715)
Supplement: Supplementary file 1 [file insects-12-00715-s001.zip › insects-1270565-supplementary.pdf]

Supplementary Materials: Samples mtCOI sequences and BLASTs scores

Sample ID	Sequence	GenBank	Seq ID	Identity	E-value
Bactericera cockerelli 1	AGCCAGTCTGATGAGATAGGGTCTCCTCCTCCGGCA<br>GGATCAAAGAAGGAAGTATTTAGATTACGGTCAGT<br>TAAAAGAATGTGATAGCTCCTGCTAGAACAGGAAG<br>AGCTAGCAAAAGAAAAATTGCAGTGATTAATACAG<br>ATCACACAAATAGTGGTATTTTTTCTAATCTACATAT<br>ACATCTTCGTATATTAATAATTGTTGTAATAAAATTG<br>ATTGCTCCTAAAATAGAAGAAATTCCAGCAAGATGT<br>AGTGAAAAAATAACTGTATCTACAGAGTATCCACTA<br>TGAAAAATAGAATTGGATAAAGGAGGATATACAGT<br>TCAACCTGTCCCTACACCTTGATCAATTAATCTACTT<br>ATAATTAACAGATACAAAGATGGAATTAGGAGTCA<br>AAATCTTATGTTATTTATTCGGGGGAAATGCAAATT<br>TTTTTTTTTACACTAGGAAAAAGACACGGTTTTTTTT<br>TTGTGGGGGGGGGAACCTAATAAAATTTCTGT	gi|102114<br>3420|gb| KU501214<br>.1|	KU501214<br>.1	97.356	0
Bactericera cockerelli 2	GCGGTATGATGAGATAGGGTCTCCTCCTCCGGCAG<br>GATCAAAGAAGGAAGTATTTAGATTACGGTCAGTT<br>AAAAGACTGTGATAGCTCCTGCTAGAACAGGAAGA<br>GCTAGCAAAAGCTAAATTGCAGTGATTAATACAGAT<br>CACACAAATAGTGGTATTTTTTCTAATCTACATATAC<br>ATCTTCGTATATCAATAATTGTTGTAATAAAATTGAT<br>TGCTCCTAAAATAGAAGAAATTCCAGCAAGATGTAG<br>TGAAAAAATAGCTGTATCTACAGAGTATCCACTATG<br>AAAAATAGAATTGGATAAAGGAGGATATACAGTTC<br>AACCTGTCCCTACACCTTGATCAATTAATCTACTTAT<br>AATTAACAGATACAAAGATGGAATTAGGAGTCAAA<br>ATCTTATGTTATTTATTCGGGGAAATGCTTTTTTTTT<br>TTTTGAGAAGAGAGGAAAAAACCTTTTTTTTTTTTTG<br>GGGGGGAAACCGATTATTATTTTTCTTTTGGTAC	gi|102114<br>3420|gb| KU501214<br>.1|	KU501214<br>.1	97.115	0
Bactericera cockerelli 3	ATTTTTTTATGAAAATAGGGTCTCCTCCTCCGGCAG<br>GATCAAAGAAGGAAGTATTTAGATTACGGTCAGTT<br>AAAAGTATTGTGATAGCTCCTGCTAGAACAGGAAG<br>AGCTAGCAAAAGTAAAATTGCAGTGATTAATACAG<br>ATCACACAAATAGTGGTATTTTTTCTAATCTACATAT<br>ACATCTTCGTATATTAATAATTGTTGTAATAAAATTG<br>ATTGCTCCTAAAATAGAAGAAATTCCAGCAAGATGT<br>AGTGAAAAAATAGCTGTATCTACAGAGTATCCACTA<br>TGAAAAATAGAATTGGATAAAGGAGGATATACAGT<br>TCAACCTGTCCCTACACCTTGATCAATTAATCTACTT<br>ATAATTAACAGATACAAAGATGGAATTAGGAGTCA<br>AAATCTTATATTATTTATTCGTGGAAATGCAATTTTT<br>TTTTT	gi|102114<br>3420|gb| KU501214<br>.1|	KU501214<br>.1	98.551	0

Bactericera cockerell i 4	GKRGSGGCTGATGAKATAGGGTCTCCTCCTCCGGCAGGATCAAAGAAGGAAGTATTTAGATTACGGTCAGTTAAAAGTATTGTGATAGCTCCTGCTAGAACAGGAAGAGCTAGCAAAAGTAAAATTGCAGTGATTAATACAGATCACACAAATAGTGGTATTTTTTCTAATCTACATATACATCTTCKTATATTAATAATTGTTGTAATAAAATTGATTGCTCCTAAAATAGAAGAAATTCCAGCAAGATGTAGTGAAAAAATAACTGTATCTACAGAGTATCCACTATGAAAAATAGAATTGGATAAAGGAGGATATACAGTTCAACCTGTCCCTACACCTTGATCAATTAATCTACTTATAATTAACAGATACAAAGATGGAATTAGGAGTCAAAATCTTATATTATTTATTCGTGGAAATTTTTTTTTTT	gi|1021143420|gb|KU501214.1|	KU501214.1	98.526	0
Nesidiocoris tenuis Spain_1	TGTGAAAAGGGGTATCCACCATAAATAAATTTACAATTTATCGCCTAAATTAATTCAGCCACCTTTTCATGAACAAATGATTATTATCAACCAATCACAAAGACATTGGTACTTTATACTTCATATTAGGTATATGAGCAGGCATGTTAGGAACATCTATAAGATGGATTATTCGAATTGAATTAGGAATACCCGGGTCATTTATCGGAAATGATCAGACATATAATGTAATAGTAACTGCTCACGCATTTGTAATAATTTTTTTCATAGTTATACCTATTATAATCGGAGGGTTCGGAAATTGATTAGTTCCTCTAATGATTGGGGCCCCTGACATAGCTTTCCCTCGAATRAATAATATAAGATTTTGACTTCTACCCCCTTCACTAACTCTTCTAACTATAAGAAGTATAGTAGAAAGA	gi|385252529|gb|JQ806057.1|	JQ806057.1	99.291	0
Nesidiocoris tenuis Spain_2	TGTGAAAAGGGGTATCCACCATAAATAAATTTACAATTTATCGCCTAAATTAATTCAGCCACCTTTTCATGAACAAATGATTATTATCAACCAATCACAAAGACATTGGTACTTTATACTTCATATTAGGTATATGAGCAGGCATGTTAGGAACATCTATAAGATGGATTATTCGAATTGAATTAGGAATACCCGGRTCATTTATCGGAAATGATCAGACATATAATGTAATAGTAACTGCTCACGCATTTGTAATAATTTTTTTCATAGTTATACCTATTATAATCGGAGGGTTCGGGAAATTGATTAGTTCCTCTAATGATTGGGGCCCCTGACATAGCTTTCCCTCGAATAAATAATATAAGATTTTGACTTCTACCCCCTTCACTAACTCTTCTAACTATAAGAAGTATAGTAGAAAGAGGGGCAGGGACAGGATGAACAGTATACCCTCCTCTGTCGGGGAATCTAA	gi|385252529|gb|JQ806057.1|	JQ806057.1	98.943	0
Nesidiocoris tenuis Spain_3	TGTGAAAAGGGGTATCCACCATAAATAAATTTACAATTTATCGCCTAAATTAATTCAGCCACCTTTTCATGAACAAATGATTATTATCAACCAATCACAAAGACATTGGTACTTTATACTTCATATTAGGTATATGAGCAGGCATGTTAGGAACATCTATAAGATGGATTATTCGAATTGAATTAGGAATACCCGGGTCATTTATCGGAAATGATCAGACATATAATGTAATAGTAACTGCTCACGCATTTGTAATAATTTTTTTCATAGTTATACCTATTATAATCGGAGGGTTCGGAAATTGATTAGTTCCTCTAATGATTGGGGCCCCTGACATAGCTTTYSCTCGAATAAATAATATAAGATTTTGACTTCTACCCCCTTCACTAACTCTTCTAACTATAARAAGTATAGTAGAAAGAGGGGCAGGGACAGGATGAACA	gi|385252529|gb|JQ806057.1|	JQ806057.1	98.874	0

Nesidiocoris tenui s Spain_4	GTGAAAAGGGGTATCCACCATAAATAAATTTACAATTTATCGCCTAAATTAATTCAGCCACCTTTTCATGAACAAATGATTATTATCAACCAATCACAAAGACATTGGTACTTTATACTTCATATTAGGTATATGAGCAGGCATGTTAGGAACATCTATAAGATGGATTATTCGAATTGAATTAGGAATACCCGGGTCATTTATCGGAAATGATCAGACATATAATGTAATAGTAACTGCTCACGCATTTGTAATAATTTTTTTCATAGTTATACCTATTATAATCGGAGGGTTCGGAAATTGATTAGTTCCTCTAATGATTGGGGCCCCTGACATAGCTTTCCCTCGAATAAATAATATAAGATTTTGACTTCTACCCCCTTCACTAACTCTTCTAACTATAAGAAGTATAGTAGAAAGAGGGGCAGGGACAGGATGAACAGTATACCCTCCTCTGTCGGG	gi|385252529|gb|JQ806057.1|	JQ806057.1	99.353	0
Nesidiocoris tenuis Texas_1	TGTGAAAAGGGGTATCCACCATAAATAAATTTACAATTTATCGCCTAAATTAATTCAGCCACCTTTTCATGAACAAATGATTATTATCAACCAATCACAAAGACATTGGTACTTTATACTTCATATTAGGTATATGAGCAGGCATGTTAGGAACATCTATAAGATGGATTATTCGAATTGAATTAGGAATACCCGGGTCATTTATCGGAAATGATCAGACATATAATGTAATAGTAACTGCTCACGCATTTGTAATAATTTTTTTCATAGTTATACCTATTATAATCGGAGGGTTCGGAAATTGATTAGTTCCTCTAATGATTGGGGCCCCTGACATAGCTTTCCCTCGAATAAATAATATAAGATTTTGACTTCTACCCCCTTCACTAACTCTTCTAACTATAAGAAGTATAGTAGA	gi|385252529|gb|JQ806057.1|	JQ806057.1	99.523	0
Nesidiocoris tenuis Texas_2	TGTGAAAAGGGGTATCCACCATAAATAAATTTACAATTTATCGCCTAAATTAATTCAGCCACCTTTTCATGAACAAATGATTATTATCAACCAATCACAAAGACATTGGTACTTTATACTTCATATTAGGTATATGAGCAGGCATGTTAGGAACATCTATAAGATGGATTATTCGAATTGAATTAGGAATACCCGGGTCATTTATCGGAAATGATCAGACATATAATGTAATAGTAACTGCTCACGCATTTGTAATAATTTTTTTCATAGTTATACCTATTATAATCGGAGGGTTCGGAAATTGATTAGTTCCTCTAATGATTGGGGCCCCCGACATAGCTTTCCCTCGAATAAATAATATAAGATTTTGACTTCTACC	gi|385252529|gb|JQ806057.1|	JQ806057.1	99.211	0
Nesidiocoris tenui s Texas_3	TGTGAAAAGGGGTATCCACCATAAATAAATTTACAATTTATCGCCTAAATTAATTCAGCCACCTTTTCATGAACAAATGATTATTATCAACCAATCACAAAGACATTGGTACTTTATACTTCATATTAGGTATATGAGCAGGCATGTTAGGAACATCTATAAGATGGATTATTCGAATTGAATTAGGAATACCCGGGTCATTTATCGGAAATGATCAGACATATAATGTAATAGTAACTGCTCACGCATTTGTAATAATTTTTTTCATAGTTATACCTATTATAATCGGAGGGTTCGGAAATTGATTAGTTCCTCTAATGATTGGGGCCCCTGACATAGCTTTCCCTCGAATAAATAATATAAGATTTTGACTTCTAC	gi|385252529|gb|JQ806057.1|	JQ806057.1	99.472	0

Nesidiocoris tenuis	Texas_4	TGTGAAAAGGGGTATCCACCATAAATAAATTTACAA TTTATCGCCTAAATTAATTCAGCCACCTTTTCATGAA CAAATGATTATTATCAACCAATCACAAAGACATTGG TACTTTATACTTCATATTAGGTATATGAGCAGGCAT GTTAGGAACATCTATAAGATGGATTATTCGAATTGA ATTAGGAATACCCGGGTCATTTATCGGAAATGATCA GACATATAATGTAATAGTAACTGCTCACGCATTTGT AATAATTTTTTTCATAGTTATACCTATTATAATCGGA GGGTTCGGAAATTGATTAGTTCCTCTAATGATTGGG GCCCCYGACATAGCTTTCCCTCGAATAAATAATATA AGATTTTGACTTCTACCCCCTTCACTAACTCTTCTAA CTATAAGAAGTATAGTAGAAAGAGGGGCAGGGAC AGGATGAACAGTATACCCTCCTCTGTCGGGGAATCT AAGA	gi|385252529|gb|JQ806057.1|	JQ806057.1	99.156	0
